# The Influence of Genetic Factors and Cognitive Reserve on Structural and Functional Resting-State Brain Networks in Aging and Alzheimer’s Disease

**DOI:** 10.3389/fnagi.2019.00030

**Published:** 2019-03-06

**Authors:** Manuela Pietzuch, Anna E. King, David D. Ward, James C. Vickers

**Affiliations:** ^1^Wicking Dementia Research and Education Centre, College of Health and Medicine, University of Tasmania, Hobart, TAS, Australia; ^2^Population Health Sciences, German Center for Neurodegenerative Diseases (DZNE), Bonn, Germany

**Keywords:** fMRI, Alzheimer’s disease, default mode network, cognitive reserve, BDNF, APOE

## Abstract

Magnetic resonance imaging (MRI) offers significant insight into the complex organization of neural networks within the human brain. Using resting-state functional MRI data, topological maps can be created to visualize changes in brain activity, as well as to represent and assess the structural and functional connections between different brain regions. Crucially, Alzheimer’s disease (AD) is associated with progressive loss in this connectivity, which is particularly evident within the default mode network. In this paper, we review the recent literature on how factors that are associated with risk of dementia may influence the organization of the brain network structures. In particular, we focus on cognitive reserve and the common genetic polymorphisms of *APOE* and *BDNF* Val66Met.

## Introduction

Recently, it was estimated that more than 47 million elderly people are affected by dementia globally (Alzheimer’s Disease International, 2009; Prince et al., 2016) and that an additional 131 million people will develop this health-challenging syndrome by 2050 (Prince et al., 2016). Alzheimer’s disease (AD), a progressive condition causing behavioral changes, memory loss, and decline in learning capacity (Anand et al., 2014), is the most common cause of dementia worldwide (Hardy, 1997). Most cases of AD occur in individuals over the age of 75, but, relatively younger individuals, including those carrying certain genetic mutations (Loy et al., 2014), may develop the disease before 65 years of age (Alzheimer’s Association, 2015).

Knowledge of the brain changes that occur in AD has increased remarkably from the late 20th century due to extensive research on a range of related neurodegenerative processes. Particular progress has been made with regard to what has been termed the pathological ‘hallmarks’ of AD – the presence of amyloid plaques and neurofibrillary tangles (NFTs) – which detrimentally affect axons, dendrites, and synapses (Vickers et al., 2000, 2016). Plaques are the result of accumulations of an abnormal form of the beta amyloid (Aβ) protein in the brain. NFTs are formed by the aggregation of aberrant tau protein (Vickers et al., 2000; Savva et al., 2009) and are more directly related to the death of neurons (Jacobs et al., 2012). Within the cerebral cortex, the earliest plaques are usually found in the neocortex, whilst initial formation of tangles occurs in medial temporal lobe (MTL) structures, such as the entorhinal cortex and hippocampus (Price and Morris, 1999). The MTL is a very important region responsible for memory formation and long-term memory (Squire and Zola-Morgan, 1991). Throughout the cerebral cortex, neurons that provide long corticocortical connections are the most prone to NFT-induced deterioration (Morrison and Hof, 1997), which may then underlie the pattern of synaptic loss seen in AD. Entorhinal-hippocampal circuits are compromised early in AD, followed by the gradual disconnection of the MTL, and then the loss of connectivity between association neocortices (Morrison and Hof, 1997). This pattern of progressive and degenerative pathology may underlie the deterioration of certain cognitive functions during aging, leading eventually to frank AD. The early pathological accumulation of Aβ has been linked to cognitive impairment and could also affect functional connectivity between spatially distant brain regions (Delbeuck et al., 2003). A summary table of studies examining functional connectivity and Aβ in healthy aging and AD can be found in Table 1. Neuroimaging is a vital component of international research collaborations (Hendrix et al., 2015) and has been used to investigate mechanisms of interrupted structural and functional connectivity underlying the course of AD (Dennis and Thompson, 2014). A better understanding of how the pathological changes in AD affect the organization of brain networks, or how these networks may respond or adapt to accumulating pathology, might offer further insights into the potential scope of functional resilience. The term resilience is described as the capability of a tissue to be resistant to damage (Cosco et al., 2017). In this respect, factors such as education and lifestyle could increase resilience by heightened connectional redundancy and/or preserving functional connections in the brain, and may ultimately delay the clinical expression of AD pathology. Indeed, studies investigating the association of education and cognitive decline in AD have found that more highly educated individuals are able to tolerate more neuropathology before the clinical expression of AD (Bennett et al., 2003), potentially because education moderates the relationship between brain pathological load and cognitive impairments (Brayne et al., 2010; Valenzuela et al., 2011), as well as functional connections (Marques et al., 2016).

**Table 1:** Studies examining functional connectivity and amyloid-beta in healthy aging and Alzheimer’s disease.

Study	Samples	Imaging measures	Main findings
Fischer et al. (2015)	CN preclinical AD (*n* = 12), Age-matched controls (*n* = 31)	DTI using tractography, measuring fludeoxyglucose-PET	CN preclinical AD (with Aβ positivity) exhibited similar white matter network changes to clinical AD as compared to controls; for instance, CN preclinical AD had more shorter paths and reduced global efficiency compared to controls.
Grandjean et al. (2014)	Transgenic mice (*n* = 38) Wild-type mice (*n* = 36)	Structural MRI, Rs-fMRI DTI	The progression of functional connectivity was disrupted in somatosensory and motor cortex in ArcAβ transgenic mice compared to wild-type mice. This decrease was noticeable even before amyloidosis in transgenic mice.
Mormino et al. (2011)	CN older (*n* = 44), AD (*n=*22)	Structural MRI, Rs-fMRI, PIB-PET imaging	Increased Aβ in CN older individuals was associated with decreased default mode network functional connectivity in multiple posteromedial regions suggesting that the accumulation of Aβ and related brain changes occurs before overt cognitive impairment.
Sheline et al. (2010b)	35 AD, 68 CN older PIB- (*n* = 24) PiB+ (*n=*20)	Structural MRI, Rs-fMRI, and Dynamic PET scan	CN people with Aβ deposition exhibited impairments in functional connectivity, particularly default mode network disruptions.
Bero et al. (2012)	Young APP/PS1 transgenic mice (*n* = 7) Old APP/PS1 transgenic mice (*n* = 7) Young wild type mice (*n* = 13) Old wild type mice (*n* = 10)	Functional connectivity optical intrinsic signal imaging	Aβ accumulation was related to decreased functional connectivity in older APP/PS1 mice compared to young APP/PS1 mice and wild-type mice. Brain regions that had more Aβ showed the most conspicuous age-related decreases in connectivity.
Hedden et al. (2009)	38 CN older adults, PIB- (*n* = 17), PiB+ (*n* = 21)	Structural MRI, fMRI, Dynamic PET	Functional connectivity was disrupted in CN older adults with Aβ positivity. Connectivity impairments related to Aβ deposition were evident between the hippocampus and posterior cingulate (default mode network regions) and associated with memory deficit.
Drzezga et al. (2011)	CN PiB- (*n=*12) CN PiB+ (*n* = 12) MCI PiB+ (*n* = 13)	Structural MRI, Rs-fMRI, fluorodeoxyglucose-PET, PiB-PET	MCI with Aβ burden exhibited hypometabolism, decrease of neuronal activity and disruption of functional connectivity in posterior brain regions (precuneus/posterior cingulate) compared to CN older adults.
Lim et al. (2013)	165 CN PIB- (*n* = 116) PiB+ (*n* = 49) BDNF Met carriers (*n* = 58) BDNF Val/Val (*n* = 107) APOE e4 (*n=*70)	Structural MRI, PET PiB imaging, Neuropsychological assessments at baseline, 18 and 36 months	BDNF Met carriers with Aβ burden positivity demonstrated an accelerated decline in memory function as well as a reduction of hippocampal volume compared to BDNF Val homozygotes.
Franzmeier et al. (2017b)	CN Aβ+ (*n* = 24) amnestic MCI Aβ (*n* = 44)	Structural MRI, Rs-fMRI, FDG-PET	Individuals with amnestic MCI with Aβ positivity and more years of education demonstrated attenuation of precuneus hypometabolism and relatively increased global frontal cortex functional connectivity.


*AD Alzheimer’s disease, Aβ Amyloid-beta, APP/PS1 Amyloid precursor protein presenilin, APOE Apolipoprotein E, BDNF Brain-derived neurotrophic factor, CN Cognitively normal, DTI Diffusion tensor imaging, FDG Fluodeoxyglucose, MCI Mild cognitive impairment, MRI Magnetic resonance imaging, PET Positron-Emissions-Tomography, PiB Pittsburgh Compound B, Rs-fMRI Resting-state functional magnetic resonance imaging.*

Studies have shown that functional connectivity is damaged or interrupted in AD (Stam et al., 2006, 2008), and, conversely, investigating the impact of AD on structural and functional networks may also provide more accurate information regarding brain connectivity and how brain regions communicate with each other (Sheline and Raichle, 2013). This review focuses on the methods with which brain connectivity is analyzed, the changes in structural and functional networks found in AD, and the role of cognitive reserve and specific genetic factors in partially determining functional brain connectivity. In this regard, potential changes in functional connectivity and resistance to pathology will involve both non-modifiable and modifiable factors that will impact on how brain systems respond to accumulating pathological burden. Hence, we discuss features of structural and functional brain networks in relation to genetic biomarkers and environmental factors linked to AD risk, progression and resilience.

## Methods to Analyze Connectivity

**FIGURE 1:**
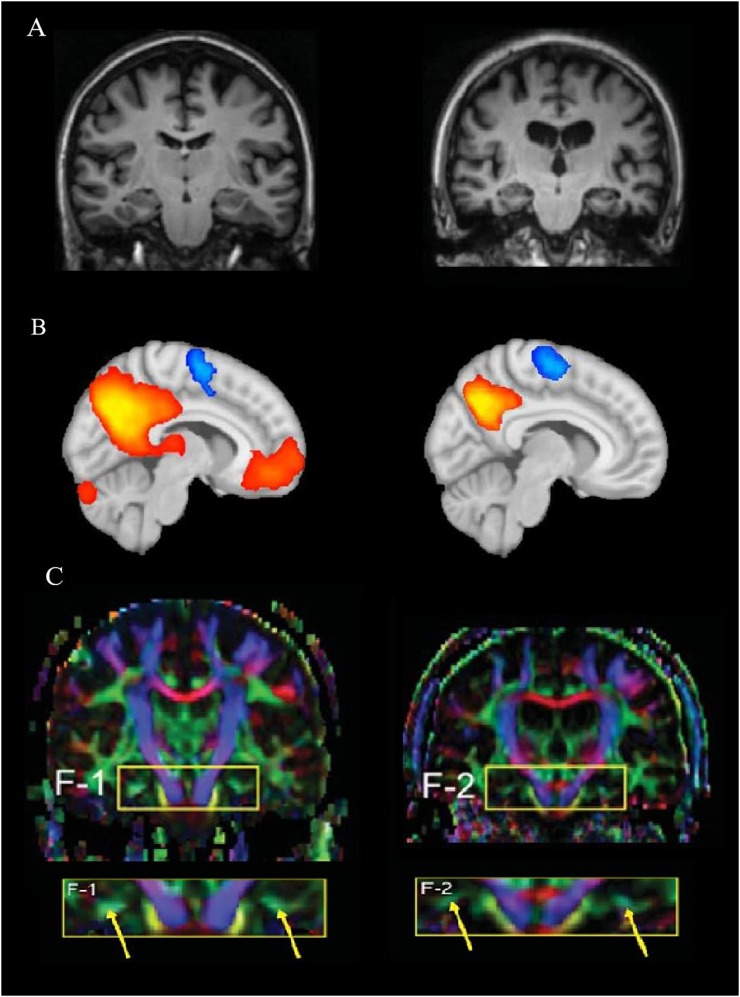
Differences among the imaging techniques, MRI, fMRI, and DTI. **(A)** A structural MRI comparison between a healthy human brain (left) compared to pathological changes in Alzheimer’s disease (AD, right; Oishi et al., 2011). **(B)** A functional MRI representing brain activation of a resting-state network in a healthy brain (left) compared to a hypothetical AD brain activation (right). The representation of the connectivity map shows how brain activity decreases with pathology within the default mode network (DMN); red/orange represents higher connectivity, while blue represents inversely correlated activity. **(C)** A comparison between a cognitively healthy woman (72 years old, left) and a woman with AD (70 years old; Oishi et al., 2011). The yellow arrows show the different color strength of the cingulum hippocampal area after DTI analysis. **(A,C)** Reprinted from Oishi et al. (2011) with permission from IOS Press. The publication is available at IOS Press through https://content.iospress.com/articles/journal-of-alzheimers-disease/jad0007.

Neuroimaging techniques (Figure 1), such as magnetic resonance imaging (MRI), have long been used to investigate anatomical connections, detect pathological alterations, and monitor the progression of neurodegenerative diseases, including AD (Figure 1A). MRI involves the generation of a strong static magnetic field to create images and to map fluctuation signals related to brain activity (Heeger and Ress, 2002). MRI also allows the quantification of brain atrophy, which can be used to distinguish normal brain aging from AD (Frankó et al., 2013). For example, a recent study found that MRI and cognitive testing in cognitively healthy individuals are useful tools for predicting the development of AD, particularly when investigating the progress from healthy cognition to the appearance of mild cognitive impairments (MCIs) after 5 years (Albert et al., 2018). The delayed presence of clinical symptoms makes it challenging to diagnose individuals in preclinical stages. Therefore, animal models could provide an opportunity to identify biomarkers of early disease (Sabbagh et al., 2013), which include insights from neuroimaging, such as gray and white matter alterations measured by diffusion tensor imaging (DTI; Weston et al., 2015).

Diffusion tensor imaging is an MRI-based neuroimaging method that measures the diffusion of water molecules, enabling the assessment of the fiber-tract structures of white matter (Jones et al., 2013; Teipel et al., 2016). This technique allows the strengths and differences of white matter tract connections in specific population groups to be compared (Jones et al., 2013) before a reduction of cognition is evident (López-Gil et al., 2014), for example between older individuals with and without AD (Figure 1C). Other structural imaging parameters that are currently used to gain further insight into the integrity of the brain over the life include intracranial volume and the presence and number of white matter hyper-intensities (Bartrés-Faz and Arenaza-Urquijo, 2011).

Functional MRI (fMRI) permits simultaneous monitoring of the activity of different brain regions while a subject is at rest or performing a task (Binder et al., 1999). In fMRI, oxygen in blood is measured through blood-oxygen-level-dependent (BOLD) signals (Ogawa et al., 1990; Heeger and Ress, 2002). Specifically, the underlying premise is that more oxygen is required for greater neuronal activity, thereby creating a signal that can be detected using fMRI (Figure 1B). Thus, it is possible to measure changes in oxygen concentration, cerebral blood flow (CBF) and volume (CBV) that are delayed by 1–2 s after MRI excitation. This is referred to as the hemodynamic response (Buxton et al., 2004). If the BOLD signal from different areas of the brain show similar and synchronized activity, it is assumed that these regions communicate with each other and transfer information, which is defined as functional connectivity (Raichle, 1998). Functional connections, defined as temporal correlations between spatially distant cortical brain regions, are revealed through fluctuations in low-frequency portions of BOLD signals (Ogawa et al., 1990). With age, functional connectivity networks gradually decrease (Dennis and Thompson, 2014), which may be important for understanding early AD or the series of brain changes that make the older brain more or less susceptible to additional disease processes.

Resting-state fMRI is an increasingly frequent method employed to study differences between various cohorts and involves the investigation of the activity of the brain while the individual is at rest and not performing a task. Resting-state fMRI can be used to determine how different brain regions operate and process information in functional space. Additional advantages are that resting-state fMRI is less demanding on the individual and easier to apply than task-related fMRI (Sheline and Raichle, 2013). The individual is instructed to not fall asleep while keeping their eyes closed in a lying position.

**FIGURE 2:**
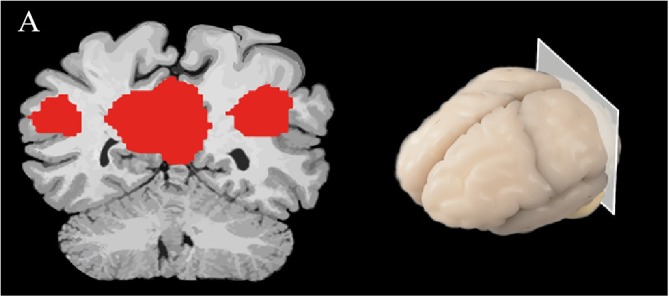
A spatial map of a brain slice is represented, demonstrating brain activity in the DMN; red represents regions that are most active while the individual is at rest.

There are a variety of approaches for analyzing resting-state fMRI. For instance, seed-based analysis (Beckmann et al., 2005) investigates the BOLD signals between the selected region of interest (seed region) and the rest of the brain (Biswal et al., 1995). In AD, the precuneus has showed decreased functional connectivity to other brain regions, such as the left hippocampus, left parahippocampus, anterior cingulate cortex and gyrus rectus, as compared to non-dementia controls (Sheline and Raichle, 2013). The investigation of simultaneous neuronal connections across the brain is called independent component analysis (ICA), and is a wholly data-driven form of analysis (Beckmann et al., 2005) (Figure 2). Using ICA-based analysis, Greicius et al. (2004) reported a decline of resting-state functional connectivity between hippocampus and posterior cingulate cortex (PCC) in the AD group compared to healthy older individuals.

**FIGURE 3:**
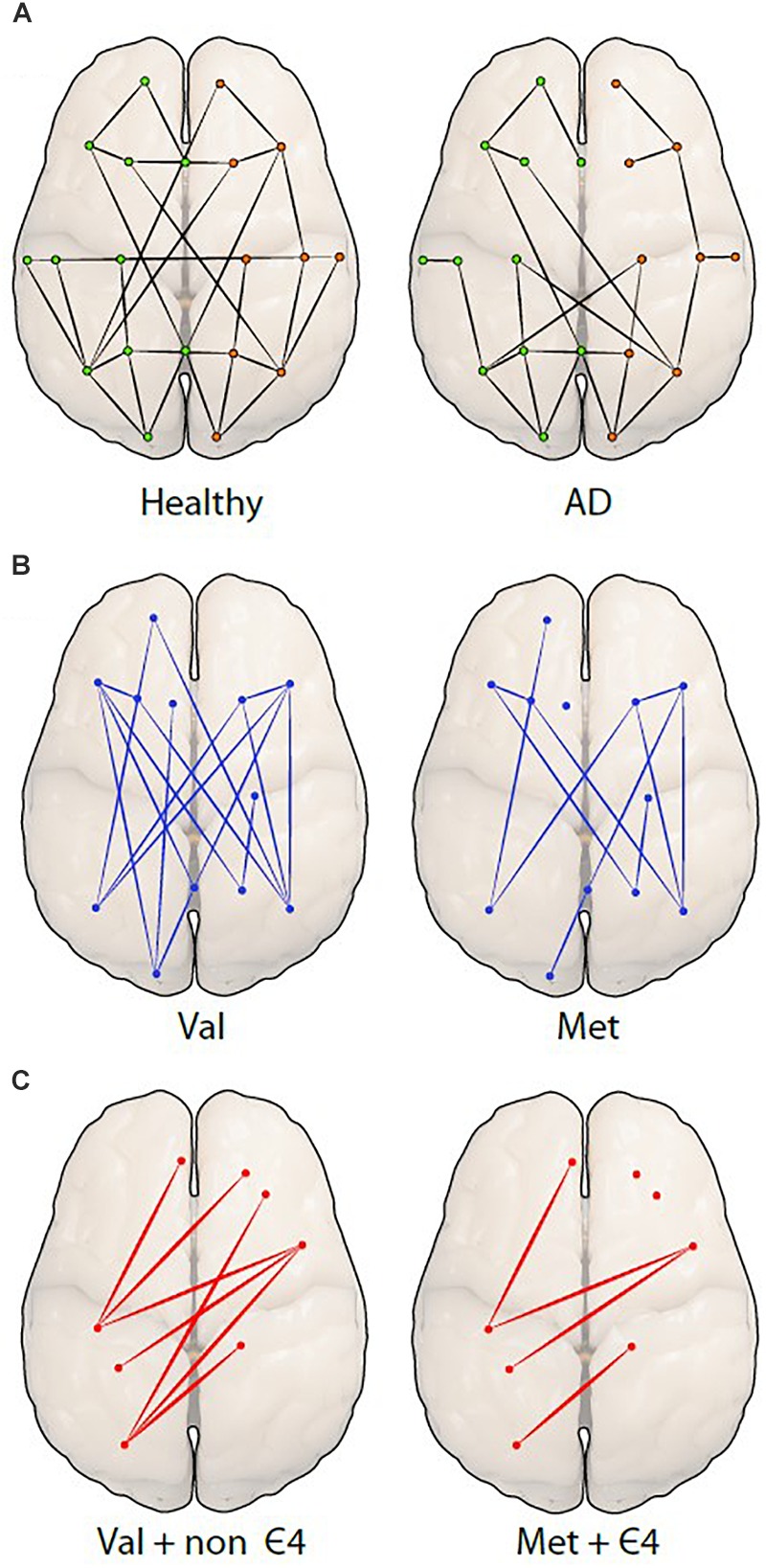
Differences among network organizations are shown using a graph theoretical approach. **(A)** A graph of a healthy person (left) is compared to a person with Alzheimer’s disease (AD; right), showing fewer connections (edges) between the spatially distant brain regions (nodes or dots) in AD. The green (left) and orange (right) dots represent hemispheres. The next two figures are hypothetical figures of the *BDNF* Val66Met polymorphism **(B)**, in which the connections are noticeably decreased in Met carriers. The last figure represents the carriage of both **(C)**, *BDNF* and *APOE* displaying a distinct reduction of edges and nodes in individuals.

Another technique used to examine resting-state functional connectivity is graph analysis, which employs a way of specifically visualizing the complex interactions in the brain (Mijalkov et al., 2017). Using graph theory, functional connectivity is represented as a series of ‘nodes’ (voxels) and ‘edges’ (correlated activity between nodes) (Watts and Strogatz, 1998; Stam et al., 2007). It has been predicted that small-world networks in human fMRI studies with low-frequency oscillation might reveal connectivity of the brain structure. A specific focus of this form of analysis in network organization is the average minimum number of edges that must be traversed between any two nodes in a brain network, referred to as ‘effective path length.’ The characteristics of small-world networks are clustering coefficient, high integration and their typical feature is shorter effective path length (Travers and Milgram, 1967; Rubinov and Sporns, 2010; Kaminski and Blinowska, 2018). Cluster coefficient is described as a measurement of nodes that are locally interconnected (Kaminski and Blinowska, 2018). This approach is particularly useful when measuring and comparing differences in structural and functional connectivity (Bullmore and Sporns, 2009), and could be used to advance our understanding of the pathology of neurodegenerative diseases (Figure 3A). A further advantage of graph theory analysis is that it makes no assumptions about how close any two nodes are in space.

## Changes in Structural and Functional Connectivity in AD

### Structural Connectivity

In AD, the loss of connections between neurons can result in other structural alterations, such as atrophy, hypometabolism, and NFT accumulation (Zhang et al., 2009). Significant atrophy in AD, identified through MRI, occurs in the posterior hippocampus and the temporal and parietal cortices, which are three of the structures that are involved in the default mode network (DMN; Greicius et al., 2003). The default mode is a network in the brain that is activated when individuals are not engaged in a task, but are spontaneously thinking of past or future events (Buckner et al., 2008). The DMN is a highly interconnected set of cortical regions that demonstrate substantial correlated activity, particularly when the attentional network is inactive (Shulman et al., 1997; Buckner et al., 2008).

Diffusion tensor imaging studies investigating white matter changes in individuals with AD have demonstrated that the disease causes a deterioration of white matter fiber bundles in the MTL (Zhang et al., 2007), which may be present years before overt episodic memory deficits (Sexton et al., 2010), impaired executive function (Reijmer et al., 2014), and other symptoms of cognitive impairment (Zhang et al., 2007; Fischer et al., 2015). Similarly, in an animal model, López-Gil et al. (2014) reported neuronal differences in structural networks of chronically hypertensive rats before the manifestation of disrupted executive functioning occurred, which may provide insights into early stages of dementia. Moreover, Grandjean et al. (2014) discovered reduced fractional anisotropy values in transgenic mice with cerebral Aβ. In cognitively healthy individuals with elevated Aβ in the brain, potentially the pathological correlate of early AD, structural changes appear similar to individuals with MCI in terms of the topology of structural network connectivity (Fischer et al., 2015). Interestingly, these individuals with high brain Aβ load despite no overt cognitive symptomatology, demonstrated increased shortest path length in white matter networks in the absence of major neurodegenerative features such as atrophy or reduction of cortical glucose (Fischer et al., 2015).

Finally, the structural networks (or nodes) of individuals with AD who possessed fewer connections (or edges) were more susceptible to global disruption of white matter tracts than individuals with more connections (Daianu et al., 2015). In addition, a rat transgenic model bearing mutant human amyloid precursor protein (APP) and presenilin genes also demonstrated a reduction of local and global efficiency, as well as less clustering as compared to non-transgenic rats (Muñoz-Moreno et al., 2018). Moreover, Muñoz-Moreno et al. (2018) found alterations in the right medial PFC in these transgenic rats, while in human studies, the right medial frontal cortical areas in AD indicated a decline in nodal efficiency compared to healthy controls (Lo et al., 2010). In summary, changes in structural connectivity could be useful in predicting the degradation of white matter bundles, as well as the strength of functional connectivity networks (Greicius et al., 2009).

### Functional Connectivity

Performance within many domains of cognitive function decreases slowly with age, but, importantly, higher cognitive performance has been correlated with increased functional connectivity in older adults (Arenaza-Urquijo et al., 2013). Compared to animal studies, transgenic AD rat models require longer cognitive training to achieve the same performance as non-transgenic rats. Although the structural network was changed, these alteration did not result in functional network differences proposing associations between the capability to learn and the reorganization of functional networks in the brain (Muñoz-Moreno et al., 2018). Nevertheless, a gradual decrease in functional connectivity among the hippocampus and medial prefrontal cortex (PFC) is expected with age (Damoiseaux et al., 2016).

It has been proposed that disconnection of functional networks in the brain, such as those observed in AD, could serve as critical markers for the presence of early stages of neurodegenerative diseases, particularly with regard to the abnormal accumulation of Aβ in the brain (Stam et al., 2007; Zhang and Raichle, 2010). Resting-state studies have reported a decrease in functional connectivity in healthy older individuals with Aβ burden in the posteromedial regions, ventral medial PFC, right angular gyrus, and the left middle and superior frontal gyri (Mormino et al., 2011), as well as between the precuneus and left hippocampus, parahippocampus, anterior cingulate, gyrus rectus and dorsal cingulate (Sheline et al., 2010a,b). Early accumulation of Aβ in older healthy individuals, particularly in the precuneus, has been suggested to result in impairment in hippocampal function (Sheline et al., 2010a,b). In contrast, Mormino et al. (2011) reported that DMN connectivity responds in a varied manner to the presence of higher Aβ deposition in older non-demented people with Aβ accumulation. Specifically, the authors found that there was increased connectivity in regions of the right dorsal PFC, left anterior medial PFC and left temporal cortices, as well as decreased DMN connectivity in several posteromedial regions, the ventral medial PFC, right angular gyrus, and the left frontal gyri (Mormino et al., 2011). Disruption within the DMN has also been found in healthy older individuals with high amyloid burden (Hedden et al., 2009). Interestingly, these healthy individuals (*n* = 38) exhibited the same amount of Aβ burden compared to half of the individuals with MCI (*n* = 46) and all individuals with AD (*n* = 35).

Such associations have also been investigated in animal models. Bero et al. (2012) demonstrated an aging-related reduction of bilateral functional connectivity in the retrosplenial cortex in wild-type mice, which could be a pre-existing biomarker for neural dysfunction due to its significant association with memory performance (Corcoran et al., 2011). Interestingly, in transgenic AD mouse model involving cortical amyloidosis, it has been shown that an age-related decrease in functional connectivity in specific brain regions is more severe in the presence of higher Aβ deposition (Bero et al., 2012). Grandjean et al. (2014) also reported reduced functional connectivity in transgenic mice, however, this reduction appeared in the early months before the accumulation of Aβ in the somatosensory and motor cortex.

A study investigating whole-brain connectivity found abnormalities in cortical hubs of the temporo-parietal cortex and precuneus/PCC in healthy mild cognitive impaired subjects with Aβ burden (Drzezga et al., 2011). In general, greater atrophy has been related to less brain connectivity (Hoffstaedter et al., 2015), but not all studies have found support for this association. For example, a study by Gili et al. (2011), reported that functional connectivity decline was not related to the amount of gray matter atrophy in the PCC in individual with MCI.

Disconnection between functional networks could be an essential biomarker for AD. For instance, individuals with AD exhibit disruption of functional connectivity between the inferior lateral temporal cortex (ITC), precuneus, right thalamus and the PCC (Zhang et al., 2009), between the left hippocampus and PCC (Sorg et al., 2007), as well as between the right hippocampus and the right and left cuneus, precuneus, and right ITC (Wang et al., 2006). This pattern of disconnection is likely associated with impairments in memory, processing speed and executive function (Damoiseaux et al., 2016). Another proposed early biomarker for AD could lie in the disruptions that have been identified within the visual cortices, specifically the impairments in connectivity between the PCC and the dorsal and ventral visual pathways (Zhang et al., 2009). These changes have been suggested to lead to deteriorating visual function in AD (Zhang et al., 2009).

Small-world network analysis in AD has shown longer path length in the central, temporal, and frontal brain regions as compared to age-matched, non-demented individuals (Stam et al., 2007). Decreased local connectedness within networks, also called clustering, has also been reported in individuals with AD, and correlated with lower cognitive performance (Stam et al., 2007). This finding led Stam et al. (2007) to speculate that individuals in the early stages of AD may show relatively diminished topology of small-world networks. A recent study found support for this notion by demonstrating that individuals with MCI and AD had a longer characteristic path length compared to healthy controls (Mijalkov et al., 2017). Moreover, AD appeared to be associated with a greater number of edges connecting to a node regionally, as well as increases and decreases in the efficiency of local nodes when compared to the controls (Mijalkov et al., 2017). To understand these differences in network topology, it is necessary to account for genetic variations that might affect the organization of the brain and which may also be linked to neurodegeneration in AD (Figure 3A).

## Role of Genetic Factors Related to AD in Functional Connectivity

### Apolipoprotein E (APOE)

The inheritance of gene-related factors such as apolipoprotein E (*APOE*), in particular the *APOE* ε4 allele, is associated with an increased risk of AD (Mahley et al., 2006, This genetic polymorphism is associated with increased Aβ deposition in the brain (Mahley et al., 2009; Morris et al., 2010; Sheline et al., 2010a), possibly influencing brain functional connectivity (Mahley et al., 2009), as well as affecting cognitive functioning in older age (Wisdom et al., 2011).

Resting-state fMRI studies have reported diverging associations of *APOE* polymorphisms and functional con-nectivity in healthy individuals that may relate to the age of the sample groups (Goveas et al., 2013; Wu et al., 2016). For example, *APOE* ε4 alleles have been associated with both increased and decreased DMN functional connectivity in cognitively healthy individuals (Fleisher et al., 2009). Comparing non-demented middle-aged (50–65 years) individuals carrying the *APOE*ε4 with non-carriers, ε4 carriers showed elevated functional connectivity in the middle frontal gyrus, whilst non-ε4 carriers had greater functional connectivity in the right medial frontal gyrus (Wu et al., 2016). Conversely, Goveas et al. (2013), demonstrated decreased functional connectivity within the DMN in cognitively healthy *APOE* ε4 carriers (44–65 years of age) in the bilateral dorsomedial PFC, superior frontal gyri, and in the left hippocampus, as well as increased functional connectivity in the left lentiform nucleus and bilateral caudate. Additionally, a decrease in interhemispheric functional connectivity within the DMN was found in healthy elderly *APOE*ε4 carriers (65–80 years of age; Lu et al., 2017). Notably, most of these regions are also affected in AD, which emphasizes the significance of the involvement of the DMN in the preclinical phase of AD (Sheline et al., 2010a). More recently, Zheng et al. (2018) investigated functional connectivity in young adults who were APPs/presenilin-1/2 mutation carriers or *APOE*ε4 positive carriers relative to adults without these AD-linked genetic factors (18–35 years). Interestingly, greater functional connectivity was observed in both the *APOE*ε4 carriers and in the APP/presenilin-1/2 group as compared to healthy controls. This increased connectivity was found between the left hippocampus and the bilateral medial PFC/precuneus. Only *APOE*ε4 carriers displayed increased connectivity between the right hippocampus and the left middle temporal gyrus. Here, the authors have suggested that the ‘beneficial’ effect of *APOE* ε4 in functional connectivity in younger individuals may be due to mechanisms of compensation of cognitive disruptions, which may be detrimental as the individual ages.

Due to inconsistencies in published evidence, it is important to consider how *APOE* polymorphisms may be associated with other measures of functional connectivity. Studies that have investigated *APOE* effects in small-world networks have reported higher susceptibility of fewer functional hubs and reduced centrality in healthy older ε4 carriers compared to non-ε4 carriers (Seo et al., 2013). Regional cerebral glucose metabolism, clustering of whole-brain functional networks, and path length have all been reported to be decreased in ε4 carriers (Seo et al., 2013). However, in a study with a greater sample size of 147 cognitively normal individuals, more clustering and longer path lengths were identified in ε4 carriers when compared to non-carriers (Goryawala et al., 2015). Non-demented ε4-carriers also had more long-distance connections in the parietal and temporal lobes, whilst non-ε4 carriers exhibited more short-distance connections in the parietal and occipital lobe. Healthy older individuals with the ε4 allele also had less short-distance connections in the frontal lobe connections, while both groups showed more long-distance connections in the frontal lobe (Goryawala et al., 2015). In summary, this study found the brain networks of those carrying *APOE*ε4 to be organized into an abnormal structure when compared to non-carriers, with fewer connections in the frontal lobe and more structural long length connections, which could partially explain the negative *APOE* ε4 cognitive phenotype.

### Brain-Derived Neurotrophic Factor (BDNF)

Another genetic factor related to AD is the *BDNF* gene (Brown et al., 2014). The BDNF protein belongs to the family of nerve growth factors, which affect neurogenesis (Erickson et al., 2010) as well as long-term potentiation (LTP) and activity-dependent synaptic plasticity (Egan et al., 2003). Post-mortem studies of AD have shown that BDNF protein levels are decreased in the hippocampus, entorhinal cortex, temporal, frontal, and parietal cortex when compared to cognitively intact age-matched controls (Connor et al., 1997; Garzon et al., 2002). Lower BDNF levels may be related to volume loss in the hippocampus (Erickson et al., 2010), but this may be secondary to other pathological changes that occur in AD (Buchman et al., 2016). BDNF concentration is highly variable between individuals and is relative to physiological state; for example, after physical exercise, peripheral blood BDNF concentration is increased (Dinoff et al., 2016). A recent review supported this finding by reporting increased neurogenesis and plasticity in the hippocampus in rats and mice after treadmill exercise, which led to improved short- and long-term memory functions (Jahangiri et al., 2018).

A common single nucleotide polymorphism in the *BDNF* gene, specifically a valine-to-methionine substitution at codon 66 (Val66Met), has an influence on LTP as well as activity-dependent BDNF secretion (Egan et al., 2003). *BDNF* Val66Met has been associated with cognitive performance as well as with AD brain morphology. In particular, the *BDNF* Met gene carriers (aged 60 and older), which were in preclinical stages of AD, demonstrated reduced memory function and smaller hippocampal and temporal lobe volume as compared to Val homozygotes (Lim et al., 2013; Brown et al., 2014). Authors also observed that more physical exercise was related to larger hippocampal and temporal lobe volumes in Val homozygotes but not in Met carriers (Brown et al., 2014). Notably, in Met carriers, physical activity was linked to reduced volumes of the temporal lobe, which is likely due to more apoptotic alterations (Brown et al., 2014). Likewise, Egan et al. (2003) demonstrated that the *BDNF* Met allele is related to qualitative changes of the hippocampus, which might cause insufficient memory functioning. Studies have proposed that there might be a relationship between Aβ and *BDNF* Val66Met, in which the *BDNF* polymorphism might mediate the effects on Aβ neurotoxicity on the brain (Fahnestock, 2011). Lim et al. (2013) reported not only a faster rate of atrophy in hippocampal volume, but also a faster decline in episodic memory performance in *BDNF* Met carriers who had a high Aβ load over a 36-month period compared to healthy individuals with *BDNF* Met but low levels of Aβ. Relative to Val homozygotes with a low Aβ load, Val homozygotes with a high Aβ load also experienced reduced cognitive performance, indicating that being a Val homozygote would not necessary protect against cognitive decline (Lim et al., 2013).

In older adults with late-onset depression, *BDNF* Met carriage was associated with reduced resting-state functional connectivity between the bilateral hippocampus and cerebellum (Yin et al., 2015). *BDNF* Met carriers with late-onset depression also had reduced strong (positive) functional connectivity between the hippocampus and the temporal cortex; however, there was also evidence of increased anti-correlated (negative) functional connectivity between the hippocampus and the dorsal anterior cingulate cortex, dorsal-lateral PFC, and angular gyrus (Yin et al., 2015). Similarly, Wang et al. (2014) observed elevated functional connectivity between the dorsal lateral PFC and the anterior insula in cognitively healthy *BDNF* Met carriers. Finally, Park et al. (2017) investigated the influence of *BDNF* Val66Met polymorphism on structural networks of middle-aged healthy individuals. The authors targeted nodes and edges in their analysis and simulated manipulation of the white matter networks. They demonstrated that Val homozygotes were more robust and resistant to gray matter damage compared to Met carriers (Park et al., 2017). Studies of white matter networks determined that *BDNF* Met carriers were more susceptible to node disruptions than Val homozygotes (Park et al., 2017).

The interaction of the *BDNF* Met and *APOE*ε4 polymorphisms was investigated by Gomar et al. (2016) in healthy older adults, as well as in individuals with MCI and AD. Here, the authors found that *BDNF* Met alleles were associated with poorer cognitive performance, predominantly in memory and semantic fluency. In support, Ward et al. (2014) found decreased performance in episodic memory function in *BDNF* Met carriers, however, only in combination with carriage of the *APOE* ε4 allele, the latter perhaps representing a cumulative effect of carriage of both risk alleles. This cumulative effect may be influencing the functional brain networks and reduce connections between different brain regions. *BDNF* Met carriers may have fewer connections compared to *BDNF* Val homozygotes (Figure 3B) and *APOE* ε4/*BDNF* Met carriers may have even fewer connections compared to non ε4/*BDNF* Val homozygote carriers, which may decrease connectivity (Figure 3C).

In a separate study, *BDNF* Met*/APOE*ε4 carriers with high brain Aβ levels demonstrated a faster rate of decline over a 54-month period in verbal and visual episodic memory and language processing when compared to *BDNF* Met/non-*APOE* ε4 carriers (Lim et al., 2015). In comparison, *BDNF* *Val/*ε4 carriers with a high Aβ burden demonstrated a relatively mild reduction in cognitive functioning. In *BDNF* Met*/APOE*ε4 carriers with high Aβ load, memory deficits are detectable after 3 years, whereas it takes 10 years in *APOE*ε4*-/BDNF* Val homozygotes with a high Aβ load to reach the same clinical threshold (Lim et al., 2015). A recent meta-analysis investigated the relationship between *APOE* and *BDNF* *Val66Met* and concluded that there were more women with AD carrying the *BDNF Met* polymorphism (Zhao et al., 2018). However, no significant relationships between *APOE*ε4 carriers and *BDNF* Met carriers were identified in the overall analysis that included both men and women with AD.

*APOE* and *BDNF* polymorphisms may interact with each other and possibly influence functional connectivity. *BDNF* Met carriers with the *APOE* ε4 allele exhibited decreased brain activation in the MTL (Kauppi et al., 2014). Atrophy, particularly in the entorhinal cortex, and acceleration of AD pathology, has been linked to poor compensation mechanisms of the brain in individuals with *BDNF* Met carrying the *APOE*ε4 (Gomar et al., 2016). Ward et al. (2015b) investigated the effect of *BDNF* and *APOE* on cognitive function and cognitive reserve, the latter which is a theoretical construct where neural networks compensate for lost neurons and connections (Stern, 2002). The authors observed that the *BDNF* Val66Met polymorphism, but not *APOE* variants, moderated the relationship between executive function and cognitive reserve, in which exposure to a more cognitively enriched environment was associated with better executive functioning in Val homozygotes but not in Met carriers (Ward et al., 2015a). In another study, Ward et al. (2017) investigated the same healthy older adult sample and found that differences in executive functioning between cognitive reserve tertile groups became smaller over time in *BDNF* Val homozygotes, but cognitive reserve-related differences became more pronounced in *BDNF* Met carriers. An explanation for these results is that cognitive reserve could have varying cognitive effects depending on the *BDNF* Val66Met polymorphism (Ward et al., 2017). Altogether, experimental studies indicate that the *BDNF* polymorphism influences key neurobiological processes associated with development and activity-dependent learning (Egan et al., 2003).

## Cognitive Reserve and Brain Connectivity

It is possible that common variation in the *BDNF* gene may result in differences in the development and maintenance of structural and functional networks throughout the life course, which ultimately may be associated with either better or worse brain resilience to neurodegenerative disease processes, such as in AD. Given the role of *BDNF* in development and adult brain plasticity, it is also possible that this gene variation may have an influence on the construction of patterns of connectivity that underlie resistance to pathology, perhaps related to the theoretical construct of cognitive reserve (Stern, 2002, 2006), in which neurons are compensating for impaired and lost neurons.

Stern (2002, 2009) proposed two different kinds of reserve in relation to a brain challenged by insult and/or neurodegeneration. Brain or neural reserve, which is often referred to as the ‘passive’ model of reserve, focuses on anatomical brain structures, especially brain size and the number and architecture of neurons and synapses (Katzman, 1993). This model, later revised by Satz (1993), proposed that individuals with higher synaptic count, dendritic branching and larger brain volume should be able to withstand the loss of more neurons without functional consequence, providing compensation for the pathological changes of AD (Stern, 2009). The brain reserve model suggests that most of its capacity is established in the early years of life, usually by the age of five (Reiss et al., 1996). Nevertheless, investigations have demonstrated that brain reserve may be modifiable. For example, the brains of adult monkeys are able to form and renew cells throughout life (Eriksson et al., 1998), and human brains have also been proposed to have neurogenic capacity, particularly in the dentate gyrus (Kempermann et al., 2015).

The ‘active’ model of reserve is often referred to as ‘cognitive reserve,’ which is a hypothetical construct that relates to the functional resilience of the brain against accumulating pathological changes (Stern et al., 1999). According to the theory of cognitive reserve, brains with more complex neural networks have a higher level of inbuilt redundancy, which are subsequently able to compensate for degenerative or lost neurons (Stern, 2002, 2006). Factors such as lifetime experience, educational and occupational attainment, and socioeconomic status are posited to play a significant role in the development of cognitive reserve (Stern, 2009, 2012). For example, individuals with AD and higher cognitive reserve (education levels) had greater DMN connectivity compared to individuals with AD and lower education levels (Bozzali et al., 2015). Bastin et al. (2012) on the other hand, determined that there was more cerebral pathology and reduced activity of metabolism in the temporoparietal cortex in healthy individuals with higher education. Furthermore, although Brayne et al. (2010) found that the amount of accumulation of pathological burden in the brain was not affected by the number of years of education that an individual had completed, higher levels of educational attainment was found to be associated with a lower risk of demonstrating dementia on the background of the burden of pathology.

Lifelong engagement in cognitively stimulating activities may reduce the risk of developing dementia by 40% (Scarmeas and Stern, 2003; Valenzuela et al., 2011). In support, Jahangiri et al. (2018) noted that exercise was associated with improved memory function, as well as reduced risk of developing neurodegenerative disease in different animal models. In human studies, Larsson et al. (2017) reported that individuals with higher educational attainment had a lower risk of developing AD. Similarly, in healthy participants (50–79 years), education later in life (university study for at least 12 months) was positively associated with cognitive reserve (as estimated by current psychological assessment scores) compared to those who did not complete any further university education (Lenehan et al., 2016). Associations between education and age are evident particularly in the attention and speed processing domains (Perry et al., 2017). In line with these findings, Summers et al. (2017) found that 92.5% of individuals 50 years and older who had attended university for at least 12 months showed increased cognitive performance in domains that may be a proxy for cognitive reserve.

Stern (2009) hypothesized that individuals with AD who have higher cognitive reserve possess more flexible neural networks and will retain a higher level of cognitive performance with an increasing neuropathological load. This notion of neural flexibility could potentially be demonstrated in re-organizable functional networks of the brain observed in cognitively healthy individuals (Bosch et al., 2010). In this study of healthy older individuals, higher cognitive reserve was associated with increased brain activity in the DMN, but it was also associated with decreased brain activity in regions associated with speech comprehension. In contrast, in individuals with MCI or AD, decreased activation in the DMN and more activation in language processing in subjects was associated with higher cognitive reserve (Bosch et al., 2010).

Education and cognitive reserve have a positive effect on functional connectivity networks (Marques et al., 2016) and cognitive functioning (Bozzali et al., 2015). There is evidence that high cognitive reserve levels were related to working memory, while age had a negative effect on cognition (Ward et al., 2015a). High cognitive reserve has been associated with greater functional connectivity in healthy elderly individuals (Marques et al., 2016). Arenaza-Urquijo et al. (2013) examined a cognitively healthy older population (60–80 years) and described better brain metabolism, higher gray matter volume as well as enhanced functional connectivity in individuals who had more years of early-life formal education. In particular, the authors found higher functional connectivity in regions such as the anterior cingulate cortex, right hippocampus, right PCC, left inferior frontal lobe and left angular gyrus in people with those with more education.

Marques et al. (2015) likewise examined the relationship between education and functional connectivity and found that individuals with more education had larger networks. These enlarged networks were connected to all lobes in each hemisphere and influenced functional connections in a positive way, which was predicted to moderate the effects of age on brain connectivity (Marques et al., 2015). Moreover, Marques et al. (2016) investigated whether sex and the number of years of education [used as demographic characteristics (DEM)], in 120 healthy older individuals influenced functional networks in the brain. The authors demonstrated that the DEM had a positive effect locally (in the neighborhood areas), on the strength of nodes, efficiency and on clustering coefficient, exhibiting greater communication within the networks of the occipital and parietal lobe areas. There was also a relationship found between the DEM and network transitivity indicating that individuals with more education use different neural processing (Marques et al., 2016). Network transitivity is defined as the connection between two nodes that are linked to each other via an edge in a network.

In addition, Marques et al. (2016) examined how cognitive reserve measured by educational attainment affected functional connectivity in resting state fMRI. They demonstrated that larger networks with more functional connections in the brain were related to higher cognitive reserve. Greater local efficiency and higher local clustering in the cuneus, as well as in the areas of the superior and middle occipital lobe were related to higher levels of cognitive reserve (Marques et al., 2016). The inferior temporal gyrus is predicted to have a significant role for cognitive reserve, because of its betweenness centrality and nodal strength, which demonstrated a positive correlation with cognitive reserve. The fraction of all shortest paths in the network that pass through a given node is called betweenness centrality (Rubinov and Sporns, 2010). The inferior temporal gyrus is a significant hub responsible for recognition and visualization of words and numbers (Grotheer et al., 2016), which are important functions involved in cognitive reserve networks (Marques et al., 2016). Finally, global efficiency, which is “a measure of functional integration” (Marques et al., 2016), was greater in individuals displaying higher cognitive reserve compared to individuals with lesser cognitive reserve.

Colangeli et al. (2016) conducted a meta-analysis of whether functional brain networks were associated with cognitive reserve in healthy older adults, as well as in amnestic MCI (aMCI) and AD. Findings in all subgroups showed greater functional brain activation in the anterior cingulate in the left hemisphere while performing a cognitively stimulating task (e.g., recognition memory task). However, the cognitively healthy older adult group demonstrated greater activation in several brain regions as compared to the aMCI and AD groups. These activated brain regions included the left anterior cingulate and left precuneus, the right cingulate gyrus, and the superior frontal gyrus of the dorso-lateral PFC, all of which are susceptible to degenerative changes in individuals diagnosed with AD and aMCI (Colangeli et al., 2016).

Bozzali et al. (2015) investigated whether cognitive reserve modifies resting-state functional connectivity in healthy, aMCI, and AD individuals (mean age 74.6 years). Functional connectivity was associated with the cognitive reserve proxy, education, within the DMN. Higher functional connectivity within the PCC was associated with higher education in individuals with AD, in which education possibly initiated mechanism of compensation. Education may also have led to brain plasticity and supported the PCC from atrophying. Some of the aMCI group exhibited similar connectivity strength, however, there was no strong functional connectivity found in the healthy group (Bozzali et al., 2015).

Franzmeier et al. (2016) also demonstrated that higher global functional connectivity was present in individuals with MCI with relatively higher levels of education. Individuals with more years of education and prodromal AD were able to compensate for fluorodeoxyglucose (FDG)-PET hypometabolism in the precuneus and had greater connectivity in the left frontal lobe, as well as better performance in memory (Franzmeier et al., 2017a,b). Moreover, Franzmeier et al. (2017b) demonstrated that individuals with MCI who had higher educational attainment and high Aβ levels had a more global left frontal cortex connectivity when controlled for age and sex, whereas, in healthy individuals, global left frontal cortex connectivity was not related with metabolism in the precuneus. Negative connectivity between the left lateral frontal cortex and the DMN was also found in people with MCI who had achieved higher education (Franzmeier et al., 2017a). Perry et al. (2017) demonstrated a positive correlation between years of education and cognitive functioning (e.g., visuospatial, executive function, language) but a weak relationship between education and brain networks, especially when the brain already showed evidence of age-related changes in healthy individuals. The greatest impact in age-related alterations later in life was found in the sensorimotor networks, especially those underlying processing speed and attention (Perry et al., 2017).

In summary, education early in life and other life-long cognitively stimulating activities could be possible protectors against neurodegenerative diseases, and might bolster cognitive reserve later in life (Ward et al., 2015b).

## Conclusion

The brain is a large set of complex networks that are connected structurally and functionally. Different areas of the brain share and communicate information in functional space, creating networks. These networks can be adversely or positively influenced by various genetic and environmental factors. For instance, studies reported that *APOE* ε4 was associated with decreased functional connectivity (Lu et al., 2017) and longer path length in functional networks (Goryawala et al., 2015). However, there was also decreased path length (Seo et al., 2013) and increased functional connectivity found in healthy *APOE* ε4 carriers (Wu et al., 2016). Similarly, healthy older *BDNF* Met carriers were associated with reduced functional connectivity, while Val homozygotes showed a more robust network in the brain structure (Park et al., 2017). Cognitive activities and environmental enrichment have favorable effects on *BDNF* Val homozygotes, and over time also on *BDNF* Met carriers (Ward et al., 2017), which possibly may promote maintaining healthy cognitive functioning and reduce the detrimental effects progressing age. In general, studies provided evidence that education and cognitive reserve are associated with an increase of functional connectivity in the brain networks (Marques et al., 2016). This could potentially affect brain networks in a positive way and may mitigate and protect against cognitive impairments later in life, and hopefully delay or even prevent the onset of AD (Prince et al., 2013). Future studies should investigate whether cognitive reserve and environmental enrichment work as compensatory mechanisms to influence and alter the networks of more susceptible genetic polymorphisms to AD, such as *APOE* ε4 and *BDNF* Met carriers. Education later in life increases cognitive reserve and could provide more resistance and resilience to brain pathology. Overall, these findings indicate that the functional networks of the brain are influenced by a combination of genetic and environmental factors. An improved understanding of these relationships is vital in order to fully grasp how neurodegenerative changes affect brain function, but also to determine how cognitive resilience to neurodegenerative changes may be promoted.

## Author Contributions

The manuscript of this review was prepared, formalized, and developed by MP. AK and DW assisted with refinement. JV contributed to the development and refinement of the manuscript.

## Conflict of Interest Statement

The authors declare that the research was conducted in the absence of any commercial or financial relationships that could be construed as a potential conflict of interest.
